# Management of mandibular angle and body fractures using miniplates and 3D plates

**DOI:** 10.6026/973206300200605

**Published:** 2024-06-30

**Authors:** Abhishek Balani, Praveen Saroj, Vinay Kharsan, Abhishek Karan, Heena Mazhar, Arunima Awasthy

**Affiliations:** 1 Department of Oral and Maxillofacial Surgery, New Horizon Dental College and Research Institute, Sakri, Bilaspur, Chhattisgarh, India

**Keywords:** Three dimensional plate, conventional miniplates, fractures, angle, body, mandible

## Abstract

Mandibular angle fractures have the greatest recorded rate of postoperative complications of any mandibular location and hence they present an especially difficult task for surgeons. Therefore, it is of interest to compare the conventional miniplates and three dimensional (3D) plates in management of mandibular angle fracture and body fractures.60 patients with isolated non-comminuted mandibular angle fractures and body fractures were randomly assigned into two groups by lottery. Utilizing Champy's osteosynthesis standards, group one (n = 30) received treatment with 2-mm standard miniplate and group two (n = 30) had treatment with open reduction and internal fixation utilizing 2-mm 3D locking stainless steel plates. The mean operative time was greater in conventional miniplate category as compared to three dimensional plates. Need for postoperative occlusion correction was lesser n 3 dimensional plate category. The incidence of postoperative infection was comparable in both categories. Incidental tooth damage was lesser in three-dimensional plate's category three-dimensional locking plates are an alternate strategy that has a comparable result profile to miniplates.

## Background:

The majority of fractures of mandible are mandibular angle fractures. Automobile accidents and attacks or altercations comprise two of the most common cause of fractures at mandibular angle region [1,2]. There are two primary theories as to why fractures are frequently linked to the mandibular angle. The first explanation is because the mandible's cross-sectional area is smaller than that of its adjacent segments [3,4]. The second factor weakening the area is the existence of third molars, especially those that are impacted. Considering mandibular angle fractures have the greatest recorded rate of postoperative complications of any mandibular location, they present an especially difficult task for surgeons [5,6]. Among all fractures of mandible, angle fractures have the highest rate of complications and are frequently difficult to treat. Furthermore, there is on-going debate on the best course of action for treating angle fractures [7,8]. In order to guarantee complete stability, angle fracture treatment regimens have traditionally included rigid fixation with intraoperative maxillomandibular fixation (MMF) [9,10]. But in more recent times, the application of precise intraoperative MMF as a supplement to internal fixation has grown in favour, and non-compression miniplates have become increasingly common [11,12]. Mandibular body fractures typically happen around the distal aspect of the mandibular canine and an imaginary line that represents the masseter muscle's anterior attachment site [13,14]. Body fractures can be classified into two types (favourable and unfavourable) based on the direction of the fracture line and the impact of muscle distraction on the fracture fragments [15,16]. Whereas the muscular distraction causes the bony fragments in favourable fractures to pull together, the muscle forces in unfavourable fractures cause the bony segments to shift [17,18]. Numerous muscles, including the masseter muscle, temporalis muscle, and medial pterygoid muscle, generate forces, which make the fracture undesirable [19,20]. In superomedial guidance, these muscles divert attention from the proximal bone section. Furthermore, it is possible that the mylohyoid and anterior belly of the digastric muscles contribute to the posterior and inferior displacement of the portions [21, 22]. The techniques used for managing mandibular fractures have been greatly improved throughout time. Both previous and newer techniques have been improved upon. There are now two methods for fixing mandibular fractures: semi-rigid fixation, as suggested by Champy *et al.* [8], and rigid stabilization, as recommended by Spiess [12]. Both methods have drawbacks: semi-rigid stabilization cannot ensure fracture stability, and rigid fixing makes it challenging and time-consuming to adjust the plate to the bone [14- 17]. These limitations might be circumvented using a three-dimensional (3D) plate [15-18].The idea of 3D miniplates, whose form depends on the quadrangle's role to serve as geometrically stable framework for support, was created by Farmand and Dupoirieux [15].With a 2 x 2 whole square plate and a 3 x 2 or 4 x 2 hole rectangular plate, the basic form is quadrangular [12-16]. The plates are positioned in accordance with Champy's ideas on the bone. An internal Mini-Locking-System was created in association with the AO/ASIF-Institute to address the drawbacks of loosening hardware and the requirement for flawless adaption of the conventional miniplate system [13-17]. The locking screw slides into the threaded plate holes and locks in place upon insertion. Researchers state that locking plate systems have certain benefits over other plate systems [17-21]. The locking system has the following potential benefits: (1) strengthens construct stability; (2) reduces the possibility of stripped screw holes; (3) reduces the possibility of a screw backout and the ensuing reduction loss;(4) when installed with power, offers a positive stop for locking screws; (5) facilitates plate adaptation by lowering the requirement for exact anatomic plate alignment with the underlying bone; and (6) keeps the plate in place relative to the bone, preserving reduction throughout surgery [21, 22, 23, 24-25]. The fractured mandibular portions are given 3D stability by the three-dimensional miniplate as they heal [16- 22]. A locking device prevents occlusal inconsistencies or screw slippage that could cause changes in bone alignment. In order to control mandibular fractures, three-dimensional locking plates have been devised with the hope of combining the benefits of both systems and overcoming their respective drawbacks [12- 19]. Therefore, it is of interest to compare the conventional miniplates and three dimensional (3D) plates in management of mandibular angle fracture and body fractures.

## Methods and Materials:

A prospective, randomized, clinical trial was carried out over 2 years

## Qualifications for inclusion:

Patients with isolated non-comminuted mandibular angle fractures and body fractures falling within the age range of 18 to 50 years were included, regardless of gender.

## Criteria for exclusion:

Patients who had a reduced mandibular vertical length between the bottom border of the mandible and the root apex of teeth assuming that the 3D plate would not suit a vertically short jaw. Patients with mixed dentition with preoperative infections at the location of the fracture are excluded.

## Patient's distribution:

Following eligibility assessment, sixty patients (n = 60) were randomly assigned into two groups by lottery. Utilizing Champy's osteosynthesis standards, group one (n = 30) received treatment with 2-mm standard miniplate and group two (n = 30) had treatment with open reduction and internal fixation utilizing 2-mm 3D locking stainless steel plates. Patients were given injections of cefotaxime 2 g intravenously (i.v.) as a precautionary antibiotic one hour prior to surgery, under general anesthesia, and two times a day for five days following the procedure. All hygienic precautions were followed during the postoperative period. After making an intraoral transbuccal incision, reducing and identifying the location of the fracture, placing a temporary IMF, and achieving adequate occlusion, the procedure was completed. With Champy's osteosynthesis principles, fixation was performed with two standard miniplates and 2 mm x 8 mm screws, or with a 3D locking 2-mm stainless steel plate. Three-dimensional locking plates were positioned according to Farmand and Dupoirieux instructions, where vertical bars were placed parallel to the fracture line and horizontal bars remained perpendicular to it [15].

Following plate fixation, the surgical site was liberally flushed with 5% povidone-iodine and then regular saline when the occlusion was confirmed once more. IMF was eliminated. Closure was completed and hemostasis was attained. It was documented how long it took to close the wound after the incision. IMF after surgery was avoided and only recommended in cases with postoperative occlusion derangement. It was advised to follow a soft diet for six weeks following surgery. The patient underwent follow-up every three months at one-week, four-week, and three-month intervals. The study's parameters namely need for postoperative IMF, postoperative occlusion, infection, segmental mobility, wound dehiscence, requirement for plate removal, and radiological assessment during reduction and fixation, were unknown to the senior oral and maxillofacial surgeon who assessed the cases. Pain during and after surgery was recorded using a visual analog scale. The radiolucency around the screws in the radiographs and the criteria for surgical site contamination were used to determine the level of infection.

## Statistical analysis:

Student's t-test and the Mann-Whitney test were used to compare the two systems. Version 14.0 of the Statistical Package for Social Science was used to analyze the data. When the P value was less than 0.05, a 95% confidence interval was used to classify it as significant.

## Results:

The mean operative time was greater in conventional miniplate category as compared to three dimensional plates. The findings of demographic details like gender age distribution and etiology of fracture were comparable in both categories (Table 1).

Need for intermaxillary fixation was greater in 3 dimensional plate category as compared to conventional miniplates category. Need for postoperative occlusion correction was lesser n 3 dimensional plate category. The incidence of postoperative infection was comparable in both categories. Incidental tooth damage was lesser in three dimensional plate categories (Table 2). Post-operative neurosensory disturbances were greater in 3 dimensional plate categories. The proportion of vertical displacement of mandible was comparable in both categories. The bad feeling of plate postoperatively was lesser n 3 dimensional plate categories. Chewing efficiency was greater in 3 dimensional plates compared to conventional plates (Table 3).

## Discussion:

The fundamentals of the 3D Miniplates system along with locking system are the foundation of 3D locking design [5- 9]. First, bending, vertical displacement, and shearing are the main forces to be concerned about while the mandible is functional. The vertical bars in a 3D plate that join the two horizontal bars are resistant to bending stress [12-18]. More stability is provided in three dimensions resisting torsion forces, vertical relocation, flexing, and shear stresses because the plate's box structure disperse the stresses over a surface area rather than along a single line. The term "3D plate" comes from the fact that stability is thus acquired in three dimensions [14-21]. With the locking system, the screw and plate combine to form a single, stiff, functional unit that is stabilized independently of the interface between the bone and the plate. A study [2] state that it is unclear if the locking mechanism or the plate design is to blame for the 3D locking plate's greater biomechanical robustness. In this study, the positioning of the 3D locking plate took an average of 10.34 minutes shorter than the positioning of the Champy's miniplate. The outcomes of studies on 3D plate [1, 11,22] that showed a shorter average operation time were comparable to these findings. According to a study, the locking system's average duration of operation was 6.65 minutes less than that of the conventional plate/screw system. The 3D locking plate saves time over traditional miniplates because of its rapid fixation at both the upper and lower borders and its easy adaptability to bone [10-18].

Since the symphyseal injuries are subject to higher torsional strain, 3D plates offer more stability in this area according to a study [14-21].In order to assess the biomechanical performance of four distinct types of semi-rigid fixation systems that are already in use in comparison to rigid fixation systems, researchers conducted an in vitro investigation. The results of this investigation showed that 3D strut plates are more resilient to compression stresses than Champy's method [16-23]. Considering the use of 3D plates, a study [25] observed a nine percent incidence of infection, while another study observed 5.4%. Enhancing plate stability may help reduce the risk of postoperative infections, as it has been suggested that the displacement of fractured parts is a contributing cause [18-24]. In our investigation, there were no follow-up cases of plate fracture in either group. Aside from technical considerations like the material and shape of the plate, there are other surgical factors that weaken the plate and must be taken into account while diagnosing the reason of plate fracture [1-7]. The likelihood of a plate fracture is decreased because the 3D locking plate's locking technology eliminates the need for the plate to make precise contact with the bone that underneath it in all locations, negating the need for repeated plate bending for adaptation [4- 12]. Data shows that 3D locking plates to treat a mandibular body and angle fracture leads in 3D stability, a decrease in infection rates, and a faster recovery period due to the plates' easier adaptability to the bone and concomitant stabilization at the upper and lower borders [11-19].When comparing the ratio of expenses to benefits, only one 3D locking plate was less expensive than Champy's plate because there were 50% fewer screws. It could be deemed cumbersome to utilize the 3D locking miniplate system for oblique injuries and fractures affecting the mental nerve region [20-25].The additional vertical bars added to the plates to counteract torque forces may be the cause of the overabundance implant material, which is consistent with the findings of a study [19-24].While biomechanical experiments have verified the 3D plating system's adequate stability, only a few number of clinical investigations have been documented in the literature. It is still unknown how 3D miniplates are used to treat mandibular fractures. Just 6% of the 104 AO/ASIF surgeons surveyed in a published study. Employ this kind of plate [1-12]. However, the locking mechanism in 3D plates eliminates the necessity for exact adaptation and the requirement for the plate to make direct contact with the bone; as a result, 3D locking plates may now be regarded as the superior choice for the treatment of mandibular body and angle fractures [13-20].It can be assumed that the combined qualities of the three-dimensional plate and locking plate, as opposed to Champy's miniplates, will produce a superior treatment outcome if the 3D plate is not placed in an oblique fracture [11-17]. A 2-mm miniplate with 3D holes is recommended by Jain *et al.* for the treatment of mandibular fractures [1].

## Conclusion:

Data shows that three-dimensional locking plates are an alternate strategy that has a comparable to miniplates.

## Figures and Tables

**Table 1:** Mean operative time and other demographic details of study participants in both groups

	**Mean Operative Time**	**Gender**		**Age**			**Etiology**		
		**Male**	**Female**	**18-30**	**31-40**	**41-50**	**Road traffic accidents**	**Assault**	**Fall**
Group One	55.93±16.37	88	12	54	28	18	82	10	8
Group Two	44.31±4.26	89	11	55	30	15	78	12	10

**Table 2:** Details about requirement for intermaxillary fixation, post-operative occlusion, postoperative infection and incidental tooth damage in both types of interventions

	**Requirement for intermaxillary fixation**		**Postoperative occlusion**			**Postoperative infection**		**Incidence tooth damage**	
	**Needed**	**Not needed**	**No need for occlusal correction**	**Minor occlusal correction**	**Major occlusal correction**	**No infection**	**Infection present**	**No damage**	**Minor contact**
Category One	44%	56%	32	59	9	90	10	95	5
Category Two	92%	8%	82	0	18	85	15	75	25

**Table 3:** Details about Postoperative sensory disturbance, Vertical displacement of mandible, Feeling of plate after platting and chewing efficiency after 1 week in both categories

	**Postoperative sensory disturbance**		**Vertical displacement of mandible**		**Feeling of plate after platting**		**Chewing efficiency after 1 week**	
	**No disturbance**	**Disturbance present**	**No displacement**	**Displacement**	**No feeling**	**Bad feeling**	**No difficulty**	**Difficulty present**
Category One	85	15	95	5	87	13	72	28
Category Two	65	35	95	5	79	21	89	11
